# An exploratory pilot study of the effect of modified hygiene kits on handwashing with soap among internally displaced persons in Ethiopia

**DOI:** 10.1186/s13031-021-00368-3

**Published:** 2021-05-04

**Authors:** Astrid Hasund Thorseth, Thomas Heath, Andualem Sisay, Mare Hamo, Sian White

**Affiliations:** 1 Department of Disease Control, London School of Hygiene and Tropical Medicine, London, UK; 2 Action Contre la Faim, Paris, France; 3 Action Against Hunger, Addis Ababa, Ethiopia

**Keywords:** Handwashing, Soap, Hygiene kit, Internally displaced persons, Ethiopia, Conflict, Behaviour change

## Abstract

**Background:**

Internally displaced persons fleeing their homes due to conflict and drought are particularly at risk of morbidity and mortality from diarrhoeal diseases. Regular handwashing with soap (HWWS) could substantially reduce the risk of these infections, but the behaviour is challenging to practice while living in resource-poor, informal settlements. To mitigate these challenges, humanitarian aid organisations distribute hygiene kits, including soap and handwashing infrastructure. Our study aimed to assess the effect of modified hygiene kits on handwashing behaviours among internally displaced persons in Moyale, Ethiopia.

**Methods:**

The pilot study evaluated three interventions: providing liquid soap; scented soap bar; and the inclusion of a mirror in addition to the standard hygiene kit. The hygiene kits were distributed to four study arms. Three of the arms received one of the interventions in addition to the standard hygiene kit. Three to six weeks after distribution the change in behaviour and perceptions of the interventions were assessed through structured observations, surveys and focus group discussions.

**Results:**

HWWS was rare at critical times for all study arms. In the liquid soap arm, HWWS was observed for only 20% of critical times. This result was not indicated significantly different from the control arm which had a prevalence of 17% (*p*-value = 0.348). In the mirror and scented soap bar intervention arms, HWWS prevalence was 11 and 10%, respectively. This was indicated to be significantly different from the control arm. Participants in the focus group discussions indicated that liquid soap, scented soap bar and the mirror made handwashing more desirable. In contrast, participants did not consider the soap bar normally distributed in hygiene kits as nice to use.

**Conclusion:**

We found no evidence of an increased prevalence of handwashing with soap following distribution of the three modified hygiene kits. However, our study indicates the value in better understanding hygiene product preferences as this may contribute to increased acceptability and use among crisis-affected populations. The challenges of doing research in a conflict-affected region had considerable implications on this study’s design and implementation.

**Trial registration:**

The trial was registered at www.ClinicalTrials.gov 6 September 2019 (reg no: NCT04078633).

**Supplementary Information:**

The online version contains supplementary material available at 10.1186/s13031-021-00368-3.

## Background

Crisis-affected populations are at increased risk of diarrhoeal morbidity and mortality [1] and in conflict-affected settings, children under 5 years of age are 20 times more likely to die from diarrheal disease than from violence associated with the conflict itself [2]. This is because crises often force populations to be displaced to crowded, informal living environments enabling diseases to spread more easily from one person to the next. At the same time, many of the institutions, infrastructure and social support systems that would normally support health break down, resulting in decreased diagnoses and treatment and increases in disease severity. Inadequate access to water, sanitation and hygiene (WASH) remains a global challenge, but these challenges are particularly pronounced in crisis-affected regions [3–6].

The seemingly simple act of handwashing with soap (HWWS) is associated with a 23–47% reduction in diarrhoea morbidity and up to a 25% reduction in respiratory illness [7–10]. Convenient access to handwashing soap products and handwashing facilities is a crucial determinant for enabling handwashing behaviours [11, 12]. Handwashing facilities with water and soap present, act as a reminder or cue to perform HWWS at critical times. When infrastructure is lacking, the perceived psychological trade-off (for example, perceiving handwashing to be a strenuous physical endeavour) can make HWWS less likely to be performed [11]. During humanitarian crises, the determinants of handwashing behaviour may differ from stable settings, because crises typically cause considerable disruptions of cultural and habitual norms [13]. Such circumstances may compromise health-protecting behaviours, such as HWWS, due to the multitude of other challenges facing populations. However, evidence about these behavioural shifts or the determinants of handwashing behaviour during crises remains limited [3, 11, 14].

Humanitarian crises differ from stable settings in other important ways. In a crisis, humanitarian actors typically provide hygiene items to populations rather than assuming communities can provide this themselves (as is the case in stable settings) [15]. The Sphere Standards for Humanitarian Action provides a minimum list of items to be included in hygiene kits including water containers, soap for bathing, soap for laundry, a handwashing station per household or a handwashing station with soap and water at shared toilet facilities and items to aid the safe disposal of children’s faeces [16]. However, there is no standard definition of hygiene kits and the type, quantity and quality of the components vary widely between organisations [15, 17]. The items included in hygiene kits can also vary based on the context they are being distributed. This can be influenced by the feasibility of transporting or procuring items for populations that are fleeing [16], population needs (e.g. water treatment products in areas experiencing cholera outbreaks) [18, 19], population preferences around hygiene products and WASH cluster standards [15]. There is an increasing trend of distributing cash or voucher-based assistance in combination or instead of hygiene kits or products [15, 16, 20].

Hygiene kits aim to reduce the risk of disease transmission by encouraging the increased practice of hygiene behaviours at the household level. However, there is limited evidence about the acceptability of hygiene kits, the use of hygiene kit products by crisis-affected populations and the effect of hygiene kit distributions on behaviour or health outcomes [13, 15, 21–23]. The available evidence is predominantly focused upon soap and hygiene kit distributions in camp settings or cholera outbreaks, and has documented mixed results [13, 22, 24–26]. One study in Bangladesh distributed hygiene kits to cholera cases upon discharge from treatment centres and showed promising impacts on behaviour and health outcomes [24]. The majority of other studies have focused on the feasibility of distributing hygiene kits, highlighting the challenges achieving sufficient coverage of the population [23, 27]. Many of the studies of soap and hygiene kit distributions rely on self-reported measures or proxy measures of product use and behaviour [21, 27, 28], which are considered less reliable indicators of handwashing behaviour [29]. Given this current state of evidence, a recent systematic review of health interventions for emergency settings called for further research into the behaviour change potential of hygiene kit components, particularly soap [3].

Internally displaced persons (IDPs) residing outside of camps are systematically less studied due to the complexities of researching in these settings. A 2020 systematic review of all WASH literature published about crisis-affected settings, reported that only 17% related to populations residing outside of camps, and 41% relates to IDP populations [30]. This is concerning given that in 2019 there were 15.4 million more IDPs than refugees globally and an estimated 29 million IDPs live in out-of-camp settings [31]. In these out-of-camp settings, IDPs are often overlooked by governments and non-government organisations, increasing their vulnerability [31].

Our study aimed to explore the potential to increase HWWS soap at critical times among IDPs living in an out-of-camp setting using locally available and rapidly deployable hygiene kit interventions. The pilot was also designed to explore if minor modifications to the quality of hygiene kit products could make HWWS more desirable and increase the likelihood it’s practised at critical times. We tested the inclusion of a scented soap bar, liquid soap or mirrors within the hygiene kits distributed by Action Against Hunger (AAH). The soap bars tested differs from standard soap bar included in AAH’s hygiene kits in three important ways: the soap was scented (whereas their standard soap bar was not), the soap had olive oil extracts in it, which was intended to make hands feel smoother after use (according to the manufacturer) and the cost of the soap was higher than the standard soap bar. The desire to smell nice has been found to motivate HWWS and therefore this was included as an intervention in our study [32]. Our rationale for choosing liquid soap as an intervention was based on global soap usage patterns; use of soap bar in stable and higher income settings is declining, and liquid soap now accounts for 47% of personal soap use [33]. A study of perceptions of soap bars in the US found that over half of consumers found liquid soap more convenient to use than soap bars, and 48% of consumers believed germs would remain on a soap bar after use [33]. We also hypothesised that changes to the physical environment surrounding the handwashing facility could cue behaviour and make it seem more desirable and therefore result in handwashing facilities being used more frequently or for a longer duration. We distributed a mirror with the hygiene kits to be placed over the handwashing station to test this. Adding a mirror is hypothesised to make a handwashing facility more desirable [34], but its effect on handwashing behaviour has been poorly documented to date [35].

## Methods

### Study site

The research took place between September and November 2019 in Moyale District, southern Ethiopia. At the time of the research, the district faced a protracted and complex emergency due to prolonged drought and armed conflict [36]. Regular violent clashes between unidentified armed groups and security forces [36] and recurring clashes between the district’s two ethnic groups, the Borena Oromo and Garreh Somalis resulted in district-wide instability [37]. There have been repeated cholera outbreaks in the district and a significant outbreak in 2016 [38]. During the study period, local health officials reported some suspected cholera cases and an outbreak was confirmed in January 2020 [39]. The area remains a priority area for delivering life-saving health services by the Ethiopian government and aid agencies [39].

A substantial influx of IDPs had settled within and around existing villages in Moyale district. There were no official displacement camps at the time of the study. As of October 2019, an estimated 110,000 IDPs were thought to be living in the area [40]. IDPs are responsible for constructing their shelters, which are typically dome-shaped and made of wood and plastic sheeting. Over time IDPs improve their houses by adding solid mud walls and thatched roofs. The majority of the IDP population are pastoralists and typically live near their animals, including cows, goats, sheep, camels and donkeys. Water was predominantly collected from surface water sources (lakes and ponds) and public taps, standpipes and boreholes. Water collection often included an extended journey by foot for collection or prolonged queuing times at public water sources.

Moyale district was chosen as the study location due to the large influx of IDPs in the area, the identified need for a WASH intervention [40] that was unmet by any aid organisations and because the study partner AAH, was working in the area. During the previous year, AAH had not distributed hygiene products or conducted hygiene promotion in the area, but had been running nutritional programmes. To the best of our knowledge, no other distributions of hygiene kits had taken place in the area before the research.

### Study design, sampling and recruitment

**Fig. 1:**
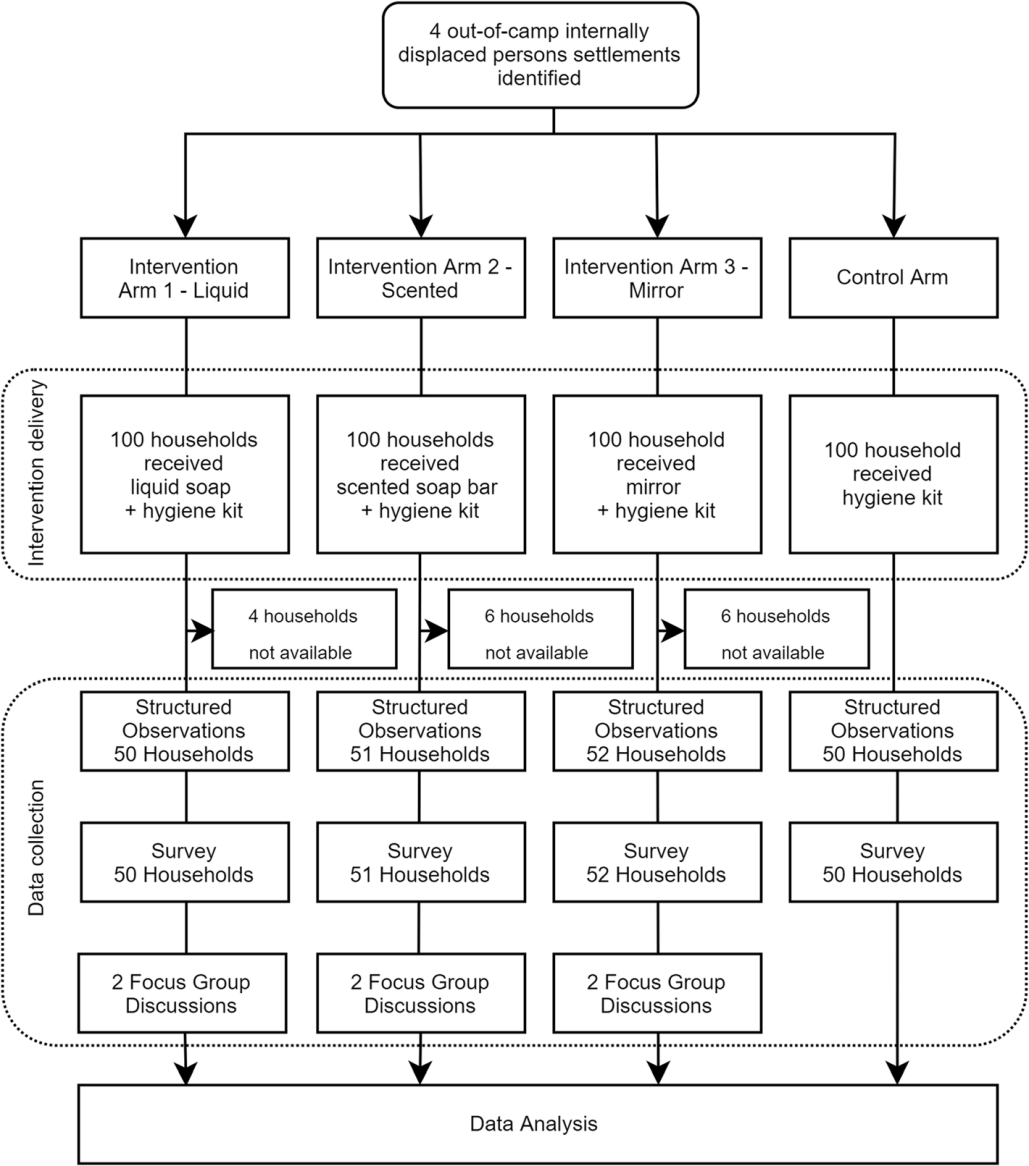
Study design of the 4-armed exploratory pilot study of the effect of modified hygiene kits on handwashing with soap among internally displaced persons in Moyale District, Ethiopia

This study was an exploratory pilot study with three intervention arms and one control arm. The villages for the study were purposefully selected by the Moyale District Disaster Risk Management Office who provided lists of 100 IDP households in each study site based on the following criteria: 1) at least 100 IDP households in the area, 2) the area was safe for the research team to work in. The other selection criteria proposed by the study team were not possible to fulfil. This included a selection process where the study team could access maps of IDP households to allow for clustering before intervention distribution and the provision of a full list of all IDP households in the study area. Due to study site constraints, randomisation at a household or cluster level was not possible. As a consequence of the lack of clustering, this study deviated from the registered trial design for the study and was not a cluster-randomised controlled trial but an exploratory pilot study. The four study arms were randomised to receive one of the three interventions or the control using a random number generator. Figure 1 shows the exploratory pilot study design. All households on the IDP household list provided by the Moyale District Disaster Risk Management office were recruited to the study. In total 400 IDP households received the hygiene kits (100 per study arm). Each study arm was located in a separate “kebele”, the smallest administrative unit in Ethiopia (similar to wards) and geographically separated by at least three kilometres. We selected a minimum of 50 households per study arm to participate in the data collection. The selected villages were home to approximately 250–1500 households [40]. However, given the dynamic population movement in the district; no reliable estimates of population size at the village level existed at the time of the study. This relatively small sample size for both distribution and data collection was determined due to logistics, budget and security constraints.

### Intervention description

**Fig. 2:**
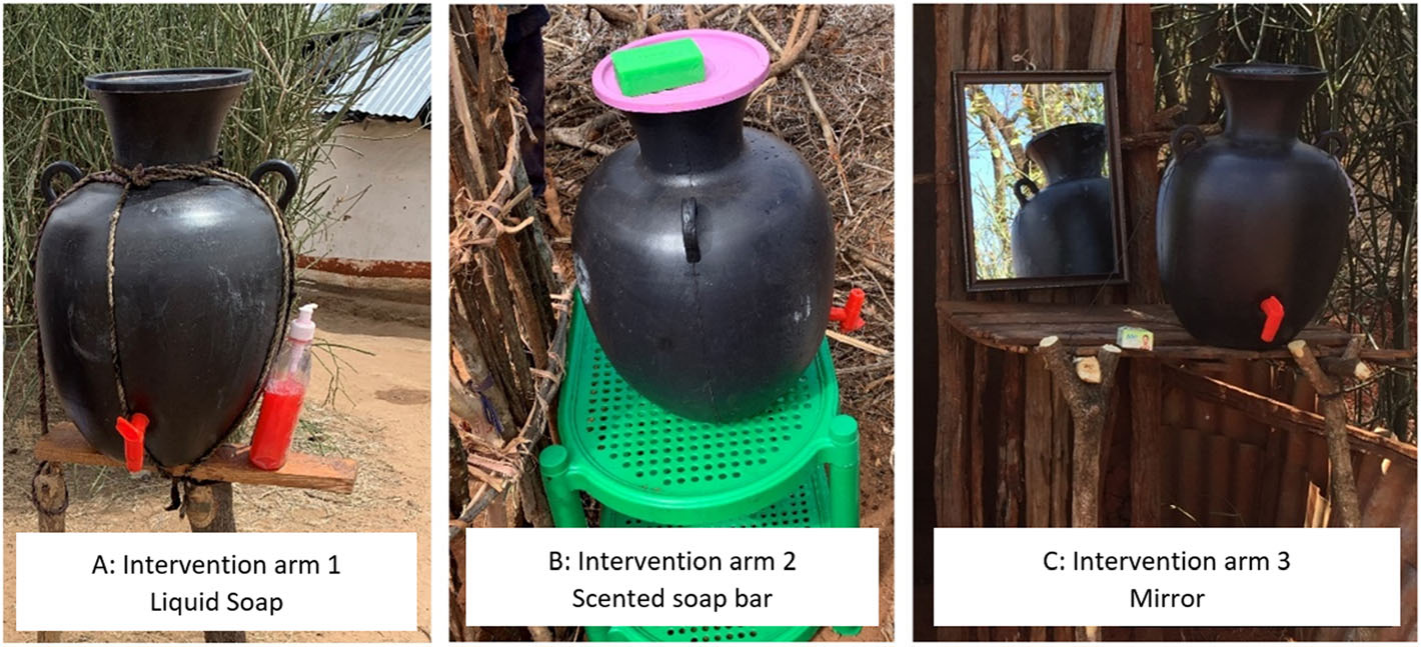
Picture **a**, **b** and **c** display the handwashing facilities distributed in all four study arms. Picture **a** shows a handwashing facility and liquid soap from IA1-Liquid, picture **b** shows a handwashing facility and a scented soap bar laying on top of the facility in IA2-Scented, and picture **c** shows a mirror and handwashing facility as distributed in IA3-Mirror

All four study arms received the interventions between the 18th to 24th September 2019. The contents of the hygiene kit received by each household were based on Sphere Standards for Humanitarian Action [16] and standards from the Ethiopian WASH Cluster. The contents were adjusted to include only items relevant to handwashing. Therefore, in this study, the hygiene kit (referred to as the “standard hygiene kit”) contained 25 × 100 g of normal soap bars (0.36 USD per soap bar), 8 × 250 g of laundry soap (0.42 USD per bar of soap) and a handwashing facility (5 USD per facility). The handwashing facility is pictured in picture A, B and C of Fig. 2. It was of 20-l capacity, had a large round body with a tap and a narrow opening on top covered by a lid. In each intervention arms, 100 households received the standard hygiene kits plus one of the three interventions: Intervention arm 1 (IA1-Liquid) received liquid soap (Pictured in Fig. 2a, 2x500ml bottles costing 1.48 USD per bottle), intervention arm 2 (IA2-Scented) received a scented soap bar (Pictured in Fig. 2b 2x250gr bars, costing 1.2 USD per 180 g bar), and Intervention arm 3 (IA3-Mirror) received a mirror (Pictured in Fig. 2c, size was 297x420mm and the price per mirror was 7.77USD). In the control arm, 100 households received the standard hygiene kit only. All items in the hygiene kit were procured from central suppliers in Addis Ababa, but they were available to purchase locally.

Implementation of the intervention was conducted by AAH hygiene promotion staff. These individuals were not involved in any other study procedures and were unaware of the planned process for the data collection on HWWS behaviour. A record was compiled of all households receiving the kits. Hygiene promoters were instructed to assist households in IA3-Mirror to hang up the mirror next to the handwashing facility. All households were encouraged to build a stand for the handwashing facility and to keep the soap near the facility at all times.

### Outcomes of interest

Our study’s primary outcome was the prevalence of handwashing with soap at critical events (after defecation, before preparing food, before eating, before serving/feeding another person food and after cleaning a child’s bottom) by IDP household members of any age. The secondary outcomes were the perceived acceptability and desirability of the interventions among the IDP population in our study area.

### Structured observations and household surveys

Handwashing events were measured through three-hour long structured observation sessions. The sessions took place from either 7:30 am to 10:30 am or 8:30 am to 11:30 am (depending on varying daily security restrictions). The research assistants were trained to document critical handwashing opportunities which were defined as 1) after using the latrine or open defecation, 2) after cleaning a child’s bottom, 3) before food preparation, 4) before eating, 5) before feeding a child or serving another person food. The research assistants captured the time of the event and if soap was used. Missed opportunities for handwashing at critical times were also captured. The structured observations were conducted at 50 households (out of the 100 that received the intervention) within each study arm. Participating households were drawn randomly (using a random number generator) from the sampling frame developed by the study team of all households who received the intervention. If the selected household could not be located on follow-up, a new household was randomly selected for data collection. A household survey was administered once the observation was concluded to capture sociodemographic data, hygiene proxy indicators [41] and perceptions of benefits of using soap. The survey respondent was the male or female head of household or another adult respondent available at the time of the survey. The survey included a spot-check which documented whether the handwashing facility was available, whether there was water in the facility and soap next to the facility. The data collection tools for structured observation and the household survey are available (see Additional files 1 and 2).

### Focus group discussions

**Fig. 3:**
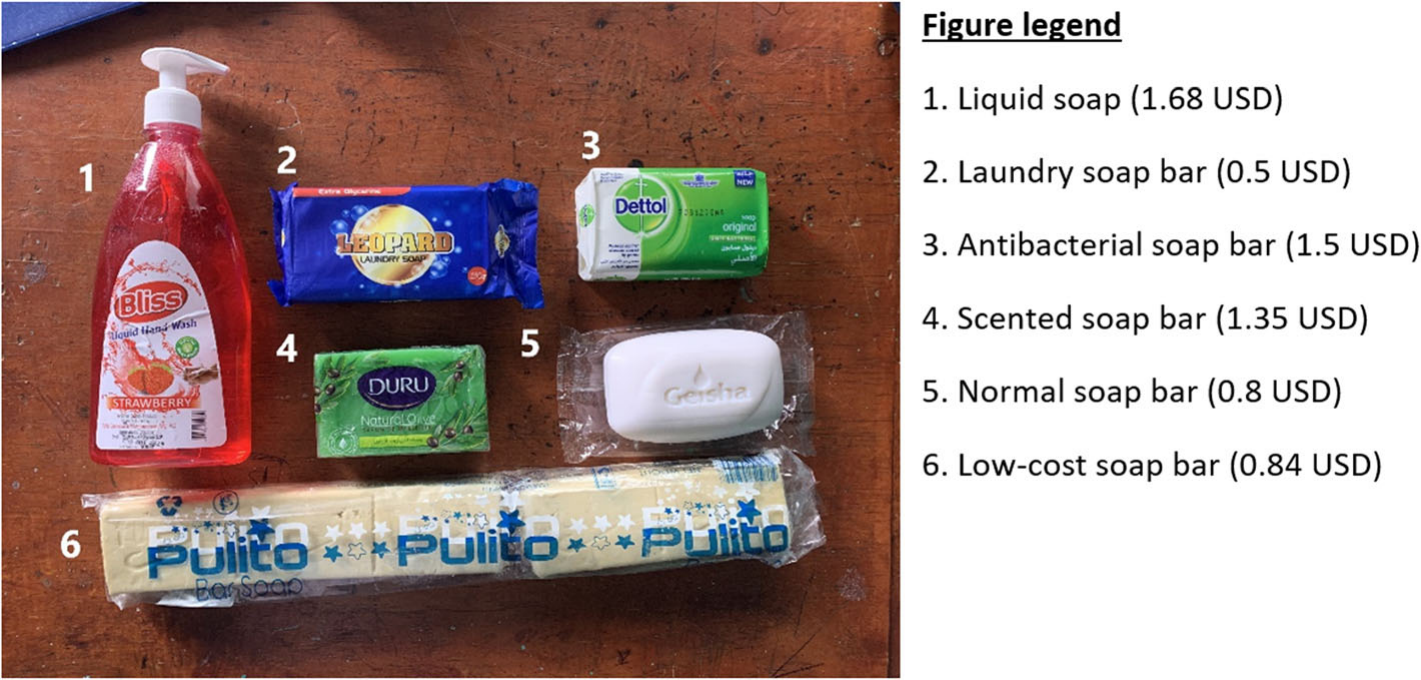
The soaps used for a soap ranking activity in focus group discussions. These soaps were purchased at a local market in Moyale District, Ethiopia

Focus group discussions (FGDs) were conducted in parallel with the quantitative data collection. Participants were randomly selected using a random number generator from a list of eligible participants. The list included households that received a hygiene kit in any of the three intervention arms but were not selected for structured observations or surveys. The FGDs aimed to explore barriers to handwashing and the acceptability and perceived usefulness of the hygiene kit products. Three FGDs with men and three FGDs with women were carried out, each with 4–8 participants. An FGD topic guide was developed (see Additional file 3). The facilitator of the FGDs first asked participants about the current challenges faced by IDPs concerning HWWS. The facilitator then introduced the participants to six different types of soap: liquid soap (as used in IA1-Liquid), a scented soap bar (as used in IA2-Scented), a standard soap bar (as included in the standard hygiene kit), an antibacterial soap bar, a low-cost soap bar and a laundry soap bar (Fig. 3). Participants then tried out each of the soaps and reflected on what they enjoyed and disliked when washing hands with each product. During the second part of the FGD, the facilitator introduced the mirror that was distributed in IA3-Mirror. Participants were asked about what they liked and disliked about the mirror and where they would hang the mirror and why.

### Consent

Written informed consent was sought from all household members over the age of 18 who participated in the observation and surveys. Information sheets and consent forms were prepared in the local language, Afaan Oromo. Parents or guardians provided consent for household members under the age of 18. Observation participants were informed that the data collectors were hoping to understand the ‘daily routines of people in the area’ and were not explicitly told that hand hygiene was being observed to minimise reactivity bias. Written informed consent was sought from all FGD participants. All FGD participants were over 18 years old.

### Data collection

Data was collected at one time-point 3–6 weeks after the hygiene kit distribution. The structured observation lasted 3 h, household surveys took approximately 20 min, and FGDs took between 45 and 75 min. FGDs took place at the kebele leader’s office. The data collection team was comprised of 16 people; 15 research assistants recruited locally by AAH and one researcher from the London School of Hygiene and Tropical Medicine (AHT). All the data collection staff were women. The research assistants received one and a half-day training by AHT on the study methods and then practised structured observation within the classroom and in a pilot study site. The research team was not connected to the intervention delivery. All data were captured on printed paper forms. At the end of each day of data collection, the lead author (AHT) checked all surveys and structured observation forms. If AHT found any discrepancies, the team would return to the household the following day to correct any inconsistencies. One member of the data collection team acted as a field supervisor and conducted spot checks of research assistants during data collection for quality control.

### Data management and analysis

Data from structured observations and surveys was double entered into Microsoft Excel and cleaned. The data were checked to identify discrepant entries against original paper surveys, and consistency checks were completed. Descriptive analysis was conducted on observational data and survey data in Stata 16 (StataCorp 2015, College Station, TX, USA). Bivariate analysis (chi-square) was used to compare intervention arms with the control arm. This deviated from the original study protocol that described regression analysis, as the data collected was not clustered due to study constraints. Handwashing prevalence was calculated as the percentage of events at which hands were washed with soap before or after a critical time (after defecation, before preparing food, before eating, before serving/feeding another person food, after cleaning a child’s bottom).

FGDs were recorded, transcribed and translated from Afaan Oromo to English. The transcripts underwent thematic analysis informed by the methods outlined by Braun and Clarke [42] and conducted with the aid of NVivo 12 (QSR International, Doncaster, Victoria, Australia). An inductive approach to identifying themes was used based on the topics covered by the FGD topic guide. This included barriers to handwashing with soap and the use of mirrors in the household. Ranking data from the FGD were summarised according to gender and analysed descriptively.

### Ethics statement

The research received ethical approval from the London School of Hygiene and Tropical Medicine Ethics Review Committee (Ref: 17604) and Oromia Regional Health Bureau (Ref: BEFO/11BTP4/79/2011). The study was also approved by the Disaster Risk Management Office and Health Office at the zonal level (Borena) and district level (Moyale) through face-to-face meetings with the study coordinator and AAH representatives.

## Results

### Sociodemographic characteristics of households that participated in structured observation and survey

In total, 400 households received a hygiene kit, of which 203 households were enrolled for structured observations and surveys. Despite the study areas being selected for their similarity, we found variation within population demographics. The control arm was a mixture of people of the Borena and Gabbra ethnic groups. In contrast, the populations in the intervention arms consisted entirely of people from the Borena ethnic group. IA3-Mirror was located 13 km from the main road in an area more affected by drought and flooding, while the other study arms were located along or within 1 km of the main road. Six of the randomly selected households in IA3-Mirror were not available for data collection as floods hindered data collectors from reaching the households. Four households in IA1-Liquid and six households in IA2-Scented were not available on follow-up because ongoing economic hardships, drought and conflict had caused them to move on. Unfortunately, the variations between study arms were not identified before the study due to security limitations in accessing the sites.

Animal ownership was over 90% in the three intervention arms, but only 54% in the control arm. The control arm results showed slightly lower educational attainment rates and household income. People in the control arm spent more time queuing to access water compared to the intervention arms. The majority of participants in all study arms had received no formal education. The mean number of people per household was similar across all study arms. All households in IA1-Liquid and the control arm were Muslims, while IA2-Scented and IA3-Mirror participants were Protestants, Muslims or practiced Wakefata (a local religion). The majority of respondents were displaced due to conflict, but 8 and 30% of respondents in IA2-Scented and IA3-Mirror respectively were displaced due to drought (Table 1).

**Table 1:** Sociodemographic data and household characteristics of the four-armed exploratory pilot study in Moyale, Ethiopia

Variable	Control arm *n* = 50	Intervention Arm 1: Liquid *n* = 50	Intervention Arm 2: Scented *n* = 51	Intervention Arm 3: Mirror *n* = 52
Number of people per household, mean (SD)	6.94 (2.65)	6.52 (2.56)	7.22 (2.15)	6.60 (2.62)
Number of children < 5 per household, mean (SD)	1.4 (1.01)	1.72 (0.86)	1.86 (1.51)	1.15 (0.89)
Respondents education, % (n)				
No education	80% (39)	62% (31)	63% (31)^a^	73% (38)
Primary school completed	16% (8)	26% (13)	24% (12)^a^	21% (11)
Secondary school completed	4% (2)	10% (5)	10% (5)^a^	6% (3)
Higher education completed	0% (0)	2% (1)	2% (1)^a^	0% (0)
Household Income per week (ETB), mean (SD)	189.29 (212.85)	219.79 (223.98)^a^	269.36 (272.53)^a^	222.06 (286.11)
Animal ownership (owning at least one domestic animal such as cow, camel, donkey, goat, sheep or chicken), % (n)	54% (27)	92% (46)	98% (50)	98% (51)
Religion, % (n)				
Muslim	100% (50)	100% (50)	61% (31)	46% (24)
Wakefata (local religion)	0% (0)	0% (0)	33% (17)	38% (20)
Protestant	0% (0)	0% (0)	4% (2)	15% (8)
No religion	0% (0)	0% (0)	2% (1)	0% (0)
Reason for displacement, % (n)				
Conflict	100% (50)	98% (49)	74% (37)^a^	56% (28)
Drought	0% (0)	0% (0)	8% (4)^a^	30% (15)
Other^b^	0% (0)	2% (1)	18% (9)^a^	14% (7)
Water collection duration (round trip) in minutes, mean (SD)	103 (77.15)	74 (60.19)	56 (65.54)	102 (71.29)
Water available per person in the household in litres, mean (SD)	14 (5.8)	13 (6.04)	12 (5.09)	14 (5.01)

^a^Percentages estimated from smaller denominators than those shown at the top of the table due to unanswered questions/missing values

^b^Other reasons for displacement included moving for job opportunities or family reasons

### Availability of handwashing facilities, soap and water

Table 2 presents the results of the household survey. Sixteen households out of the 203 households surveyed, did not have the handwashing facilities available during the follow-up visit. Among the households with the handwashing facility present during the follow-up visit, 88% of facilities contained water. Soap presence at the handwashing facility (any type of soap) was highest in the control arm (66%) while in IA1-Liquid, IA3-Mirror, and IA2-Scented soap were present in 44, 42 and 27% of households, respectively. During the distribution, households were encouraged to build a stand for the handwashing facilities. 83% of all households had constructed a stand, which were built from locally available materials such as wood. In IA3-mirror, 77% of households had the mirror hung alongside the handwashing facility at the point of follow-up. The presence of soap in the household was high across all study arms (96–100%).

**Table 2:** Results from a household survey on hygiene props and infrastructure from the four study arms in an exploratory pilot study in Moyale, Ethiopia

Variable	Control arm *n* = 50	Intervention Arm 1: Liquid *n* = 50	Intervention Arm 2: Scented *n* = 51	Intervention Arm 3: Mirror *n* = 52
Hygiene Proxy indicator (Handwashing facility with soap and water present), % (n)	64% (32)	44% (22)	25% (13)	40% (21)
Handwashing facility available on premises, % (n)	96% (48)	92% (46)	88% (45)	92% (48)
Water available at handwashing facility, % (n)	92% (44)^b^	93% (43)^b^	87% (39)^b^	83% (40)^b^
Soap available at handwashing facility, % (n)	66% (33)^b^	44% (22)^b^	27% (14)^b^	42% (22)^b^
Constructed a stand or other mechanism to raise the facility off the ground, % (n)	92% (44)	91% (42)^a^	87% (39)	83% (40)
Mirror available by handwashing facility, % (n)	0% (0)	0% (0)^a^	0% (0)	77% (37)
Soap available in household, % (n)	98% (49)	100% (50)	96% (49)	98% (51)
Types of soap available in household, % (n)				
Liquid soap	16% (8)	92% (46)	22% (11)	27% (14)
Scented soap bar	26% (13)	26% (13)	51% (26)	17% (9)
Laundry soap	72% (36)	66% (33)	63% (32)	81% (42)
Normal soap bar	88% (44)	70% (35)	76% (39)	71% (37)
Number of households reporting that they have enough soap to meet their family’s needs, % (n)	45% (22)^a^	52% (26)	45% (23)	48% (25)
Number of households reporting that soap is affordable for them, % (n)	41% (20)^a^	51% (25)^a^	52% (26)^a^	42% (22)

^a^Percentages estimated from smaller denominators than those shown at the top of the table due to unanswered survey questions/missing values

^b^Percentages estimated from the total number of handwashing facility present in the respective study arm

### Reported benefits of soap

Table 3 summarises the open-ended survey question responses “What are the benefits of soap.” Despite the distribution of hygiene kits, 45–54% of households across all study arms, reported they felt their family did not have sufficient access to soap and that soap was not affordable for them. When asked about the advantages of soap, most respondents reported that handwashing with soap could protect health and prevent disease (Table 3). A few respondents mentioned diarrhoea as a disease that HWWS can prevent. Respondents also listed cleanliness and comfort as advantages of HWWS.

**Table 3:** Advantages of soap listed by participants in the household survey in the four-arm exploratory pilot study in Moyale, Ethiopia

Advantages listed by respondents, % (n)	Control arm *n* = 50	Intervention Arm 1: Liquid *n* = 50	Intervention Arm 2: Scented *n* = 51	Intervention Arm 3: Mirror *n* = 52
To keep healthy	48% (24)	44% (22)	35% (18)	48% (25)
To remove dirt and maintain cleanliness and hygiene	34% (17)	42% (21)	53% (27)	31% (16)
To remove germs and protect against disease in general	34% (17)	48% (24)	51% (26)	46% (24)
To feel comfortable	8% (4)	4% (2)	8% (4)	2% (1)
To prevent antibiotic resistance	6% (3)	4% (2)	0% (0)	4% (2)
To prevent diarrhoea	4% (2)	2% (1)	6% (3)	0% (0)
To prevent malnutrition	2% (1)	2% (1)	0% (0)	2% (1)
To reduce absence from school	0% (0)	2% (1)	0% (0)	0% (0)
Don’t know	0% (0)	2% (1)	4% (2)	0% (0)

### Observations of handwashing

In total, 1458 opportunities for handwashing were observed by our research team (Table 4). Out of those opportunities, HWWS was observed only 218 (14%) times. HWWS prevalence is presented in (Table 4). IA1-Liquid had the highest prevalence of HWWS at critical times for HWWS. In this study arm, HWWS prevalence was 20%, but the difference was not indicated significantly different from the control arm (*p* = 0.348). In IA3-Mirror and IA2-Scented, HWWS prevalence was 11 and 10%, respectively, which was lower than in the control arm, indicating that distribution of mirrors and scented soap may have had a negative effect on HWWS prevalence. Exploratory statistical analysis suggests that this negative effect may be significant when compared to the control arm (*p* = 0.005 in IA2-Scented and *p* = 0.018 in IA3-Mirror).

**Table 4:** Observed handwashing behaviour at all critical times (after defecation, before preparing food, before eating, before serving/feeding another person food, after cleaning a child’s bottom)

Study Arm	Total number of observed possibilities for handwashing with soap	Handwashing with soap prevalence % (n)	***P***-value^**a**^
Control arm (*n* = 50)	362	17% (63)	ref
Intervention Arm 1: Liquid (*n* = 50)	409	20% (82)	0.348
Intervention Arm 2: Scented (*n* = 51)	385	10% (40)	0.005
Intervention Arm 3: Mirror (*n* = 52)	302	11% (33)	0.018

^a^Pearson Chi-square test

### Reported barriers to handwashing from focus group discussions

A total of 33 people participated in the six FGDs. When asked about current barriers to HWWS the most common challenge was the affordability of soap. Participants made it clear that knowledge was not the problem as most people knew about the importance of handwashing to protect them against disease and maintain their health.

*“Everyone now knows that it’s important to wash our hands with soaps, but affording it [soap] is the problem” (Woman, FGD2)*

*“In the old times, the problem was illiteracy. Nowadays though, everyone including the kids have the knowledge [about handwashing]. But people are different, in that some are tidy while others don’t care a lot about cleanliness. But I can generalise and say the main problem is the lack of money for soap affordability.” (Man, FGD1)*

*“There are variety of challenges, among which affording soap is an issue. People also don’t buy soaps on a regular basis in the same way they buy other home goods when they run out of it. So people also don’t look at soaps as a priority” (Man, FGD3)*

In addition, to affordability forgetting to do HWWS or only doing it when absolutely necessary were mentioned as reasons for not washing hands regularly. Some people reported only washing their hands when they were visibly dirty or when participants had been in contact with chemicals such as paint.

*“Some cannot afford soaps. The other factor is people’s style of life. Some are not used to washing with soap after using the toilet, they don’t remember to wash their hands with soaps after normal routines except when we deal with some rare activities where the need of using soap become a necessity, like after painting.” (Man, FGD3)*

IDPs shared that humanitarian organisations sometimes provide soap in hygiene kits and do hygiene promotion in the area. The irregularity of distributions appears to have created variations in handwashing behaviour, with populations often resorting to handwashing with ash or not handwashing at all, when distributions ceased. In addition, IDPs mentioned the long distances from their houses to shops and markets as barriers to purchasing soap regularly.

*“We do not get soap distributions regularly. We used to wash our hands properly when the supplies were given to us, but once they were done with the distributions, we could not go out and buy soap because of money issues.” (Man, FGD4)*

Water scarcity was frequently raised, with participants explaining that water was prioritised for other household tasks rather than HWWS.

*“In this zone when water becomes scarce, people don’t even wash their faces, let alone washing hands, so water shortage could be a reason” (Man, FGD1)*

*“Due to drought, famine, and conflict in our area, there is a water and money shortage which means we don’t have enough water for handwashing and money for affording soap, even though we have the knowledge about cleanliness.” (Man, F1)*

### Ranking of different types of soap by focus group discussion participants

Table 5 summarises the results from the soap ranking activity, in which FGD participants were asked to rank each soap against nine criteria. Participants were asked to only consider using the soap for handwashing, rather than other purposes. The scented soap bar came out the highest overall, ranking first or second for both women and men for five criteria; desirability, pleasantness, long-lasting, ‘A soap I would like to use’ and water saving. Participants from one FGD (F1) remarked that they enjoyed the scented soap bar’s smell and that they had not seen a green bar of soap before. However, one participant said that a nice smelling soap bar might be a ‘waste’ in their community because they regularly touch and come into contact with animals that have an unpleasant smell. Men and women both found the liquid soap easy to use, and believed that the antibacterial soap was the most effective in killing germs. However, these soaps were ranked inconsistently in other categories. The low-cost soap bar was the most familiar to the participants, as it was available to purchase in most local shops and markets, and it ranked consistently low. It was ranked as the soap that used the most water, was least pleasant to use, and is consumed the quickest. Men and women generally ranked soaps similarly but had a mixed attitude on liquids soap’s ability to be water-saving. On this criteria, men considered liquid soap to be the most water-saving while women considered it to be the soap that wasted the most water. There were mixed attitudes towards the use of laundry soap for handwashing. Laundry soap was ranked highest by women as the soap that would last the longest, however, the women did not find this type of soap easy to use.

**Table 5:** Summarised results from soap ranking activity where FGD participants were asked to rank different types of soap against a list of criteria describing various qualities of the soap (1 = the highest ranking and 6 = the lowest ranking)

	Desirability		Pleasantness		Long lasting		Familiarity		Something that I really would want to use		A soap the kebele leader would be likely to use		Effective at killing germs		Easy to use		Water saving	
Gender	F	M	F	M	F	M	F	M	F	M	F	M	F	M	F	M	F	M
Liquid soap	5	1	3	3	4	2	5	4	3	3	6	3	3	2	1	1	6	1
Scented soap bar	1	2	1	1	2	1	2	5	1	1	4	6	6	3	5	3	1	2
Normal soap	3	3	2	4	3	4	4	3	2	2	2	5	4	4	4	2	2	5
Antibacterial soap	2	4	5	2	5	5	6	6	5	4	5	4	1	1	3	5	4	3
Low-cost soap bar	6	6	6	6	6	6	1	1	6	6	1	1	5	6	2	6	5	6
Laundry soap bar	4	5	4	5	1	3	3	2	4	5	3	2	2	5	6	4	3	4

### Perceptions about the mirrors based on FGD discussions

The last part of the FGD aimed to understand community perceptions towards mirrors placed in close proximity to handwashing facilities. The mirror was very well received by the participants who valued the mirror’s size, reflecting that it would allow them to see their entire bodies and not just their faces. The only thing participants listed as a concern about the mirror was that they did not think it would be affordable for them to buy themselves.

*“This mirror is big enough to show the all of my body. This is why we say it’s so good.” (Woman, FGD2)*

*“I like the way it allows me to see my whole self, what I don’t like about it is the money I lack to get such a mirror,” (Man, FGD4)*

When asked where they would place a mirror like this, most participants said a nice mirror like this should be kept inside the house. Participants expressed concerns about keeping the mirror outside because they believed that the sun’s reflection shining onto the mirror was harmful to their health. They were also concerned that it might get stolen or that children or animals might break it.

*“When it is sunny, the mirror gives out a reflection which is not good for our health. It might get stolen too, cattle might break it also” (Man, FGD3)*

*“It should not be kept outside because it might get broken, it is meant to be inside the house.” (Man, FGD3)*

The majority of participants said they would not want to keep the mirror by the handwashing facility, as this was often located near the toilet, some distance from the house.

*“Firstly, that place is at a distance from our house. Secondly, children might just grab it away, the other factor is that our toilet has no suitable wooden place where we can hang the mirror.” (Woman, FGD7)*

Nonetheless, participants did see that there could be some benefits by keeping the mirror next to the handwashing facility. Some participants said keeping the mirror close to the toilet would allow them to identify dirt and make cleaning themselves an easier task. Some also reported that if they had two mirrors, they would consider keeping one in the household and one by the handwashing facility.

*“Yeah it has a benefit and that is that after toilet usage we would stand there to see which part to clean and wash our hands and our face.” (Man, FGD4)*

*“It shows me the cleanliness of my body, for example, after toilet usage, it shows me whether I have gotten rid of the dirt or not.” (Man, FGD1)*

*“It can show you dirt. Had we had other extra mirrors, we would spare one for that spot.” (Woman, FGD4)*

## Discussion

Our study could not detect an effect of providing modified hygiene kits compared to households who received the standard hygiene kits on handwashing behaviour in IDP populations. The HWWS prevalence data indicate a slightly higher prevalence of HWWS at critical times for the households that received liquid soap in addition to the standard hygiene kit. However, the prevalence was not significantly different to HWWS prevalence in the control arm. The standard soap bar distributed in hygiene kits, was not considered very desirable to use by the participants of the FGD. Instead, liquid soap was considered the easiest type of soap to use. It is possible that the increased prevalence of handwashing with soap observed within IA1-Liquid occurred because liquid soap together with a dedicated handwashing facility helped to cue behaviour at the right time and make it more convenient for the population to practice. This was particularly the case for handwashing after using the toilet, given that most families chose to locate their handwashing facilities near the toilet.

The scented soap bar was generally well-liked by FGD participants and considered desirable and pleasant to use. However, prevalence of HWWS was significantly lower in IA2-Scented compared to the control arm. In FGDs, participants reported that they had never seen a green bar of soap before, and it is possible that this new, foreign type of soap caused participants to use it more sparingly or prioritise it for purposes other than HWWS. In refugee camp setting, reliance on soap distribution may have led to refugee households using soap sparingly, not knowing when the next distribution might occur [13]. Participants also reported concern that using a nice smelling soap would be “wasted” as the smell would not last long as they frequently interact with animals.

In IA3-Mirror, we found that the distribution of mirrors and the placement of these above the handwashing facility did not result in prevalence of HWWS that were higher than the control arm. Similar ‘nudges’, or environmental cues in other studies have been successful in increasing handwashing with soap after toilet use. However, most of these interventions have been tested in schools or areas where there is already good quality infrastructure and a constant supply of soap and water - something not available in the IDP settlements of Moyale District [43, 44]. Challenges hanging a mirror outside by the handwashing facility, including being afraid of theft of the mirror or it being broken by children or animals (as reported by the FGD participants). Nonetheless, in IA3-Mirror 77% of households had mirrors next to the handwashing facility at the time of the follow-up visit. This high level of use by the community and the expressed desirability for mirrors merit further studies to explore the potential impact on handwashing behaviour.

The soap products distributed in this study did not come at a much higher cost; 250 g of the soap bar normally distributed in the hygiene kits would cost 0.9 USD, while a scented soap bar of the same size cost 1.65 USD and a 500 ml bottle of liquid soap is 1.48 USD making it a feasible intervention to implement by humanitarian actors.

This study was designed without a baseline assessment of handwashing behaviour. This was due to logistical, budget and security constraints, but also encouraged by other behaviour change intervention studies that recommended no baseline observation to reduce reactivity bias [45]. Reflecting on the constraints, a baseline observation would have allowed us to more accurately comment on the overall improvement that the modified hygiene kits might have had on HWWS. It is clear that handwashing prevalence remained suboptimal at the point of follow-up and on many critical times hands were washed with water only (see Additional file 4 for handwashing prevalence disaggregated by type of event). Other studies of handwashing behaviour in refugee camps receiving regular soap distributions in Ethiopia found HWWS prevalence of 4% [26] and 19% [22]. A recent review of national survey data estimated that the prevalence of HWWS after toilet use is likely to be about 8% within the World Health Organisation African region [46]. Our study measured behaviour shortly after the distribution of hygiene kits. It is possible that over time, with the repeated distribution of soap and with an increased familiarity of the handwashing facility, behaviour may increase.

It is also possible that it was the distribution of the handwashing facility itself, rather than soap or mirrors, that made a more substantial contribution to encourage handwashing behaviour (both with water only and with soap). However, this study was not designed to measure this. In other settings, the presence of a dedicated handwashing facility has been found to increase handwashing behaviour [11]. In our study, it seemed that the population valued the handwashing facilities that we distributed, as 83% of people were willing to invest time and effort into constructing stands to make them easy to use. This may be an early indication that the provision of higher quality handwashing products encourages higher levels of ownership and maintenance among crisis-affected populations.

Our study observed a high prevalence of handwashing with water only despite the availability of soap in households (See Additional file 4). Similar findings were identified following soap distributions in a South Sudanese refugee camp [13]. In this study, 95% of participants reported that they had soap available, but hands were normally washed with water only [13]. In this research this finding may act as a reminder that distributions in the absence of hygiene behaviour change activities may only go part of the way to enabling the desired behaviour. If our intervention was combined with hygiene promotion, it may have been possible to conduct activities that emphasised the importance of using soap and that handwashing with water only doesn’t leave hands truly clean.

### Limitations

The challenges of the research setting, particularly that the area was experiencing an ongoing conflict, created numerous limitations for interpreting our work’s findings. The heterogeneity of socio-demographic characteristics across the study arms, due to the lack of randomisation and clustering, is among the reasons it is not possible to draw clear conclusions from this research. In addition, there were other visible characteristics that may have influenced the findings. For example, IA1-Liquid, IA2-Scented and the control arm were located close to the main road. In contrast, IA3-Mirror was located 13 km off the main road in an area that was more affected by drought and water availability. This water scarcity may contribute to the low prevalence of HWWS observed in this study arm. The control arm had the longest duration for water collection, and this might have biased results. The control arm was the site of a violent conflict in 2018, and households in the area still carried the scars of this conflict, with some houses damaged and many water points destroyed. Settlements in the three intervention arms did not have these visual scars of the ongoing conflict. The study sites also experienced environmental change between the delivery of the intervention and the data collection. The long-term drought was interrupted with heavy rains that caused flooding and damage in IA3-Mirror and the control arm. Participants from all study arms reported that due to the rain, members of their households spent most of the day away from the household to tend to their farms.

Another limitation of the study is the data collection methods. Structured observation can be subject to reactivity bias leading to study participants increasing the frequency of desirable behaviours such as HWWS when observed. Therefore it could falsely inflate HWWS prevalence in our study [47]. But while having its limitations, it is considered the most reliable method of studying handwashing behaviours [48] and is regarded as much more accurate than self-reported measures. We further attempted to reduce bias by only employing female observers. As female observers are considered less intimidating in most cultures and allow for reactivity bias to be minimised [49]. Additionally, household surveys and FGDs may have been affected by social desirability bias.

For others considering research of this nature in dynamic, crisis-affected settings, we would recommend including a baseline study and taking time to understand qualitatively and quantitatively the characteristics of the study settings to understand in advance how they could impact the research outcomes. The pilot study recruited 203 households for data collection. The study team could observe 13 households per day, meaning that it took 4 weeks of data collection to reach the target of 50 households per study arm. It would have been preferable for a small-scale study to have a narrower data collection period, but that was not feasible in our setting. It would have required a larger study team, which was not possible due to logistics and security constraints. To mitigate this, data were collected on a rotating schedule (1 day in IA1-Liquid, next in IA2-Scented, then IA3-Mirror, then the control arm and then back in IA1-Liquid). Given that we faced limitations in drawing conclusions from our primary outcome data, it was valuable to learn from the complementary qualitative methods, which should be included in future research in such environments.

## Conclusion

We found no evidence of increased handwashing with soap prevalence among IDPs after distribution of modified hygiene kits. However, this pilot study indicates that there is likely to be some value in understanding crisis-affected populations’ hygiene product preferences and modify hygiene kits accordingly. In our study, provision of liquid soap resulted in the highest prevalence of handwashing at critical times, and the mirror and scented soap bar that was distributed was desired by participants. Given the limitations of this research, we recommend further studies into the use of hygiene kit products prior to major changes in humanitarian practice.

## Supplementary Information

**Additional file 1.** Structured Observation Data Collection form.

**Additional file 2.** Household Survey Form.

**Additional file 3.** Focus Group Topic Guide.

**Additional file 4.** Observed handwashing prevalence across all study arms, disaggregated by action taken and critical time for handwashing (after defecation, before preparing food, before eating, before serving/feeding another person food, after cleaning a child’s bottom) in a four-arm exploratory pilot study in an internally displaced population in Moyale District, Ethiopia.

## Data Availability

The datasets used and/or analysed during the current study are available from the corresponding author on reasonable request.

## Abbreviations

FGD: Focus group discussion
IDP: Internal Displaced Person
HWWS: Handwashing with soap
WASH: Water, Sanitation and Hygiene
