# Supplementary material for: An exploratory pilot study of the effect of modified hygiene kits on handwashing with soap among internally displaced persons in Ethiopia
Source: Confl Health. 2021 May 4;15:35. doi: 10.1186/s13031-021-00368-3 (PMC8097963; doi:10.1186/s13031-021-00368-3)
Supplement: Supplementary file 1 — Additional file 1. Structured Observation Data Collection form. [file 13031_2021_368_MOESM1_ESM.pdf]

LONDON SCHOOL of HYGIENE &TROPICAL MEDICINE

ACTION AGAINST HUNGER

USAID FROM THE AMERICAN PEOPLE

Structured Observation Form

Date: __ __ / __ __ / __ __ __ __ Start time: ___: ___ End time ___:___ Kebele name: ___________ Household ID: __ __ __ Research Assistant ID: __ __

**Time**<br>H H : M M	**When**<br>1 : After using the toilet<br>2 : Before preparing food<br>3 : Before eating<br>4 : Before serving/feeding another person food<br>5 : After cleaning a child's faeces<br>6 : Other (please describe)	**Person doing event**<br>1 : Man<br>2 : Woman<br>3 : Child between 5-18 years old<br>4 : Child under 5 years old	**Action taken**<br>1 : No action<br>2 : Washed hands with water<br>3 : Washed hands standard bar soap<br>4 : Washed hands nice bar soap<br>5 : Washed hands with liquid soap<br>6 : Did not see<br>7 : Other (please describe)	**Description of event**<br>If child washed their hands, does someone help? Who?<br>If child is fed, what kind of food? (eg. breast milk, porridge)<br>If using the option "Other" please describe here
:	1 2 3 4 5 6	1 2 3 4	1 2 3 4 5 6 7	
:	1 2 3 4 5 6	1 2 3 4	1 2 3 4 5 6 7	
:	1 2 3 4 5 6	1 2 3 4	1 2 3 4 5 6 7	
:	1 2 3 4 5 6	1 2 3 4	1 2 3 4 5 6 7	
:	1 2 3 4 5 6	1 2 3 4	1 2 3 4 5 6 7	
:	1 2 3 4 5 6	1 2 3 4	1 2 3 4 5 6 7	
:	1 2 3 4 5 6	1 2 3 4	1 2 3 4 5 6 7	
:	1 2 3 4 5 6	1 2 3 4	1 2 3 4 5 6 7	
:	1 2 3 4 5 6	1 2 3 4	1 2 3 4 5 6 7	
:	1 2 3 4 5 6	1 2 3 4	1 2 3 4 5 6 7	
:	1 2 3 4 5 6	1 2 3 4	1 2 3 4 5 6 7	
:	1 2 3 4 5 6	1 2 3 4	1 2 3 4 5 6 7	
:	1 2 3 4 5 6	1 2 3 4	1 2 3 4 5 6 7	
:	1 2 3 4 5 6	1 2 3 4	1 2 3 4 5 6 7	
:	1 2 3 4 5 6	1 2 3 4	1 2 3 4 5 6 7	
:	1 2 3 4 5 6	1 2 3 4	1 2 3 4 5 6 7	
:	1 2 3 4 5 6	1 2 3 4	1 2 3 4 5 6 7	
:	1 2 3 4 5 6	1 2 3 4	1 2 3 4 5 6 7	
:	1 2 3 4 5 6	1 2 3 4	1 2 3 4 5 6 7	
:	1 2 3 4 5 6	1 2 3 4	1 2 3 4 5 6 7	
:	1 2 3 4 5 6	1 2 3 4	1 2 3 4 5 6 7	
:	1 2 3 4 5 6	1 2 3 4	1 2 3 4 5 6 7	
:	1 2 3 4 5 6	1 2 3 4	1 2 3 4 5 6 7	
:	1 2 3 4 5 6	1 2 3 4	1 2 3 4 5 6 7	
:	1 2 3 4 5 6	1 2 3 4	1 2 3 4 5 6 7	
