# Supplementary material for: An exploratory pilot study of the effect of modified hygiene kits on handwashing with soap among internally displaced persons in Ethiopia
Source: Confl Health. 2021 May 4;15:35. doi: 10.1186/s13031-021-00368-3 (PMC8097963; doi:10.1186/s13031-021-00368-3)
Supplement: Supplementary file 2 — Additional file 2. Household Survey Form. [file 13031_2021_368_MOESM2_ESM.pdf]

SURVEY FORM

Date: ____ /____ /________ Research Assistant ID: __ __ Kebele name: _____________

1. General (ASK QUESTION G1, G2 and G3 before you start observation)

1.1 Household ID: __ __ __

1.2 How many people live in the HH? (People who sleep and eat here every day. No visitors)

....

1.3 How many children under 5 years old are in the family?

....

1.4 What level of education have you completed?

☐ Primary School ☐ Secondary school ☐ Higher education ☐ No education

☐ No answer

1.5 How much income (money) does your family generate per week on average?

.... birr

1.6 Do you own any animals?

☐ Goat, how many? ....

☐ Cow, how many? ....

☐ Sheep, how many? ....

☐ Donkey, how many? ....

☐ Chicken, how many? ....

☐ Camel, how many? ....

☐ No animals

2 LIFE IN MOYALE

2.1 How long have you lived in this location

.... Months or .... years

2.2 If the household are IDPs, what was the reason for displacement?

☐ Drought ☐ Job opportunity
☐ Conflict ☐ Illness
☐ Other: ___________________

2.3 What is your religion?

……………………………………………………………………………………………….

2.4 Have you attended any events run by humanitarian organisations in the last 3 months where they talked about hygiene?

☐ Yes ☐ No

3 Water

3.1 How much water do you collect for the whole family per day?

☐ 20 litres
☐ 40 litres
☐ 60 litres
☐ 80 litres
☐ 100 litres
☐ 120 litres
☐ 140 litres
☐ More than 140 litres

3.2 Is the water enough for your family?

☐ Yes ☐ No

3.3 What is your main water source?

☐ Public tap, standpipe
☐ Tube well or borehole
☐ Protected well (covered)
☐ Unprotected well
☐ Rainwater (covered cistern/tank)
☐ Rainwater (uncovered cistern/tank)
☐ Water truck
☐ Cart with small tank
☐ Surface water (river, dam, lake, pond, stream, canal, irrigation channel)
☐ Bottled or sachet water
☐ Other: …………………..

3.4 How many minutes does it take for you to go fetch water at this source and come back? Include queuing in the total time.

…………… minutes and ……………. Hours

4 Sanitation/waste

4.1 Where do you defecate?

☐ Pit latrine with concrete slab (not pour flush)
☐ Pit latrine without slab (not pour flush)
☐ Bucket
☐ Bag
☐ Open defecation (no facilities)
☐ Other: ....

4.2 How many people in total use your latrine?

....

5 Hygiene

5.1 Do you have enough soap for all members of the family?

☐ Yes ☐ No

5.2 Is soap affordable for you?

☐ Yes ☐ No

5.3 Can you list some of the advantages of handwashing with soap?

....

6 Spot checks

6.1 Check if the following items were available (ask to see them)

☐ Liquid Soap ☐ Green (nice) bar soap ☐ Mirror above handwashing stand

☐ Bar soap ☐ Laundry soap ☐ Handwashing facility

6.2 Where was the soap kept (e.g. inside house, at handwashing facility or in kitchen ++)

....

Only answer 6.3 and 6.4 if there is a handwashing facility present

6.3 Was there water in the handwashing facility? (Say yes if there was water in facility during observation)

☐ Yes ☐ No

6.4 Did the household build a handwashing stand for the facility?

☐ Yes ☐ No
