# Supplementary material for: An exploratory pilot study of the effect of modified hygiene kits on handwashing with soap among internally displaced persons in Ethiopia
Source: Confl Health. 2021 May 4;15:35. doi: 10.1186/s13031-021-00368-3 (PMC8097963; doi:10.1186/s13031-021-00368-3)
Supplement: Supplementary file 3 — Additional file 3. Focus Group Topic Guide. [file 13031_2021_368_MOESM3_ESM.pdf]

TOPIC GUIDE

FOCUS GROUP DISCUSSION

Focus Group Discussion – version 1

Purpose: to understand local perceptions towards different types of soap and mirrors.

Process:

1. Potential participants should be approached individually, informed about the FGD and the consent process undertaken prior to them arriving at the FGD.
2. For the FGD there should be one research assistant who acts as the primary facilitator of the discussion and another research assistant who is the scribe and supports the primary facilitator. The scribe should capture the key things that everyone says.
3. To start, get participants to introduce each other and say their favourite food, colour or a particular skill they have. These are all fairly neutral questions and are preferable over more personal topics given the experiences people may have recently been through.
4. Then explain the rules of the focus group these should include the following:
    a. There are no right or wrong answers to anything we discuss today and it is important that we respect the opinions of others in the group.
    b. In this discussion we are interested in hearing everyone's opinion. So we will encourage you all to take turs speaking and you should try not to talk over or interrupt other people while they are speaking.
    c. We want you to feel comfortable sharing your opinion with us and so we would ask that all of you agree to not share what we discuss here with those outside the group after we finish.
5. Start by asking the participants whether there are any factors that make it challenging for them and their families to wash their hands with soap. Even if they personally find it easy get them to think about factors that might make it difficult for some other people in the camp.
6. Introduce participants to 5 different hand cleaning products (ideally the type of soap that is normally distributed, different types of bar soap, scented soap, liquid soap, and laundry soap). Provide a basic explanation of how each product should be used and how it works. Ask participants to list the good and bad things about each product.
7. Set up some hand cleaning stations and get people to try each of the products (in different orders) as each person tries each product get them to think about what they like and don't like, how their hands feel and how their hands smell after using the product.
8. Bring all the products back to the centre of the room. Explain that you would like participants to rank the products according to different criteria.
   The scale parameters we will assess include:
    a. Undesirable – desirable
    b. Unhygienic - Hygienic
    c. Unpleasant – pleasurable
    d. Consumed quickly - Long-lasting
    e. Strange – familiar
    f. Something that I really wouldn't want to use – something that I really would want to use

    g. Something that the village chief is not likely to use – something that the village chief is likely to use
    h. Not very effective in killing germs - Very effective in killing germs
    i. Hard to use – easy to use
    j. Water wasting – water saving.
9. Explain to the group that we will now move onto the second part of the discussion which will be about mirrors. Ask participants whether they have a mirror at home. Where in their house do they keep it? Why? Who uses it? What type of mirrors do they own?
10. Introduce the mirror used in the study. What do they like/dislike about this mirror? If they were given this mirror where would they keep it? Would they consider placing the the mirror near the handwashing facility? If the mirror was placed above the handwashing facility what benefits might there be? Are there any bad things about keeping it near the handwashing facility? Are there any issues with keeping a mirror outside? Do you think that people would spend longer or shorter at the handwashing station if there was a mirror there?
11. At the end of the FGD thank the participants for their time, explain how the information they shared will help us improve the product and see if they have any further questions.

Focus Group Discussion – version 2

Purpose: Assessing the acceptability and response to motivational stories.

Process:

1. Potential participants should be approached individually, informed about the FGD and the consent process undertaken prior to them arriving at the FGD.
2. For the FGD there should be one research assistant who acts as the primary facilitator of the discussion and another research assistant who is the scribe and supports the primary facilitator. The scribe should capture the key things that everyone says.
3. To start, get participants to introduce each other and say their favourite food, colour or a particular skill they have. These are all fairly neutral questions and are preferable over more personal topics given the experiences people may have recently been through.
4. Then explain the rules of the focus group these should include the following:
    a. There are no right or wrong answers to anything we discuss today and it is important that we respect the opinions of others in the group.
    b. In this discussion we are interested in hearing everyone's opinion. So we will encourage you all to take turs speaking and you should try not to talk over or interrupt other people while they are speaking.
    c. We want you to feel comfortable sharing your opinion with us and so we would ask that all of you agree to not share what we discuss here with those outside the group after we finish.
5. Explain to the participants that today you are going to share with them two stories. Read out the first story and use the picture-based story book to guide the process. Ask the following:
    a. What do you like about this story?
    b. What do you dislike about this story?
    c. What do you think is the main lesson to take away from this story?
    d. If you were going to tell the same story to a friend how would you tell it?
    e. Do you identify with the people in the story? How are their experiences similar or different to your own experiences?

    f. What could be changed about the story to make it seem more like your experiences?

6. Now read out the second story again using the picture-based story book to guide the process. Ask the same set of questions as above.
7. Introduce a scale to participants from 1 – 10 (this should be created with a set of prompt cards). On this scale 1 represents the least and 10 represents the most. Ask each individual to point to a card and then discuss differences of opinion. Rate each of the stories against the following criteria:
    a. How believable is Story 1? Story 2?
    b. How emotional did Story 1 make you feel? Story 2?
    c. If we came back in 6 months how likely would it be that you could remember and retell Story 1? Story 2?
    d. How well did Story 1 hold your attention? Story 2?
    e. How much does Story 1 make you want to act differently? Story 2?
8. After this last question ask about the specific ways that the story makes them want to change their behaviour.
9. At the end of the FGD thank the participants for their time, explain how the information they shared will help us improve the product and see if they have any further questions.
