# Supplementary material for: An exploratory pilot study of the effect of modified hygiene kits on handwashing with soap among internally displaced persons in Ethiopia
Source: Confl Health. 2021 May 4;15:35. doi: 10.1186/s13031-021-00368-3 (PMC8097963; doi:10.1186/s13031-021-00368-3)
Supplement: Supplementary file 4 — Additional file 4. Observed handwashing prevalence across all study arms, disaggregated by action taken and critical time for handwashing (after defecation, before preparing food, before eating, before serving/feeding another person food, after cleaning a child’s bottom) in a four-arm exploratory pilot study in an internally displaced population in Moyale District, Ethiopia. [file 13031_2021_368_MOESM4_ESM.pdf]

Table 1: Observed handwashing prevalence across all study arms, disaggregated by action taken and critical time for handwashing (after defecation, before preparing food, before eating, before serving/feeding another person food, after cleaning a child's bottom) in a four arm exploratory pilot study in an internally displaced population in Moyale District, Ethiopia.

	Control arm n=50	Intervention Arm 1: Liquid n=50	Intervention Arm 2: Scented n=51	Intervention Arm 3: Mirror n=52
**Handwashing at all key times (after defecation, before preparing food, before eating, before serving/feeding another person food, after cleaning a child's bottom), n (%)**				
Did not wash hands	38% (137)	41% (169)	49% (187)	46% (139)
Washed hands with water	45% (162)	39% (158)	41% (158)	43% (130)
Washed hands with water and soap	17% (63)	20% (82)	10% (40)	11% (33)
**After defecation, n (%)**				
Did not wash hands	21% (22)	22% (24)	33% (30)	33% (25)
Washed hands with water	48% (49)	42% (47)	48% (43)	43% (32)
Washed hands with water and soap	31% (32)	36% (40)	19% (17)	24% (18)
**Before preparing food, n (%)**				
Did not wash hands	38% (21)	55% (30)	52% (31)	57% (30)
Washed hands with water	54% (30)	29% (16)	42% (25)	42% (22)

Washed hands with water and soap	9% (5)	16% (9)	7% (4)	2% (1)
**Before eating, n (%)**				
Did not wash hands	43% (55)	53% (77)	58% (81)	41% (43)
Washed hands with water	47% (59)	36% (52)	36% (51)	53% (56)
Washed hands with water and soap	10% (13)	12% (17)	6% (8)	7% (7)
**Before serving/feeding another person food, n (%)**				
Did not wash hands	74% (35)	52% (30)	61% (37)	61% (36)
Washed hands with water	23% (11)	40% (23)	36% (22)	36% (12)
Washed hands with water and soap	2% (1)	9% (5)	3% (2)	2% (1)
**After cleaning a child's bottom, n (%)**				
Did not wash hands	14% (4)	21% (8)	24% (8)	26% (5)
Washed hands with water	45% (13)	51% (20)	50% (17)	42% (8)
Washed hands with water and soap	41% (12)	28% (11)	26% (9)	32% (6)
